# Prognostic Value of Pretreatment Modified Glasgow Prognostic Score in Nivolumab-Treated Advanced Non-Small Cell Lung Cancer

**DOI:** 10.3390/jcm15197594

**Published:** 2026-09-30

**Authors:** Sedat Biter, Mehmet Mutlu Kıdı, Yasemin Aydınalp Camadan, Şüheda Ataş İpek, Esra Asarkaya, Hatice Asoğlu, Ertuğrul Bayram, Tolga Köşeci, Berksoy Şahin, İsmail Oğuz Kara

**Affiliations:** 1 Department of Medical Oncology, Çukurova University Faculty of Medicine, Adana 01330, Turkey; suhedaatas92@gmail.com (Ş.A.İ.); easarkaya.91@gmail.com (E.A.); drhatice10@gmail.com (H.A.); ertugrulbayram84@gmail.com (E.B.); drtolgakoseci@gmail.com (T.K.); berksoys@hotmail.com (B.Ş.); iokara@cu.edu.tr (İ.O.K.); 2 Department of Medical Oncology, Seyhan State Hospital, Adana 01150, Turkey; mehmetmutlu_01@hotmail.com (M.M.K.); yaseminaydinalp23@gmail.com (Y.A.C.)

**Keywords:** modified Glasgow prognostic Score, non-small cell lung cancer, nivolumab, immune checkpoint inhibitor, systemic inflammation, prognosis, overall survival, progression-free survival

## Abstract

**Background/Objectives**: Immune checkpoint inhibitors have become a standard treatment option for advanced/metastatic non-small cell lung cancer (NSCLC); however, considerable heterogeneity exists among patients regarding treatment response and survival. This study aimed to determine whether the pretreatment modified Glasgow Prognostic Score (mGPS), a marker of systemic inflammation and nutritional status, is prognostically associated with progression-free survival (PFS) and overall survival (OS) in patients with advanced/metastatic NSCLC treated with nivolumab at a single tertiary oncology center. **Methods:** This retrospective, single-center cohort study included 130 adult patients with histologically confirmed advanced/metastatic NSCLC who received nivolumab at the Department of Medical Oncology, Çukurova University Faculty of Medicine. The pretreatment mGPS (0, 1, or 2), derived from baseline serum C-reactive protein (CRP) and albumin levels, was analyzed in relation to PFS and OS using the Kaplan–Meier method, log-rank test, and Cox proportional hazards regression. The prognostic value of mGPS was tested in multivariable Cox models adjusted for age, sex, Eastern Cooperative Oncology Group (ECOG) performance status, histology, brain metastasis, liver metastasis, and neutrophil-to-lymphocyte ratio (NLR); the proportional hazards assumption was formally tested, and the incremental discriminative contribution of mGPS was assessed using bootstrap resampling. **Results:** Among 130 patients, 83.8% received nivolumab as second-line therapy. Pretreatment mGPS was 0, 1, and 2 in 40 (30.8%), 55 (42.3%), and 35 (26.9%) patients, respectively. Median OS was not reached, 9.7 months, and 3.0 months across mGPS 0, 1, and 2, respectively; corresponding median PFS values were 10.2, 4.8, and 3.0 months (log-rank *p* < 0.001 for both endpoints). After adjustment for age, sex, ECOG performance status, histology, brain metastasis, liver metastasis, and NLR, each one-point increase in mGPS remained associated with worse OS (adjusted HR 2.38; 95% CI 1.64–3.46; *p* < 0.001) and PFS (adjusted HR 1.81; 95% CI 1.32–2.48; *p* < 0.001), independent of the covariates included in the model. The proportional hazards assumption held for mGPS in both models. Addition of mGPS to the clinical-variables-only model was associated with higher model discrimination (Harrell’s C-index: 0.676 to 0.737 for OS and 0.620 to 0.666 for PFS), a difference confirmed as statistically significant by bootstrap resampling (2000 iterations; 95% CI for the difference excluding zero, *p* < 0.001 for both endpoints). **Conclusions:** Pretreatment mGPS is a simple, readily available marker that shows a robust prognostic—rather than treatment-predictive—association with both OS and PFS in patients with advanced/metastatic NSCLC treated predominantly as second-line nivolumab monotherapy. Its addition to established clinical variables was associated with a statistically significant, though modest, improvement in prognostic discrimination. These findings require prospective, multicenter validation, including in first-line immunotherapy settings, before mGPS can be recommended for routine clinical risk stratification.

## 1. Introduction

Lung cancer remains the leading cause of cancer-related mortality worldwide, and non-small cell lung cancer (NSCLC) accounts for approximately 85% of all cases [1]. A substantial proportion of patients present with advanced or metastatic disease at the time of diagnosis. The introduction of immune checkpoint inhibitors (ICIs) targeting programmed cell death protein-1 (PD-1) and its ligand (PD-L1) has fundamentally transformed the treatment landscape of advanced NSCLC, providing durable, long-term survival in selected patient subsets [2,3]. Nivolumab, a fully human IgG4 anti-PD-1 monoclonal antibody, was among the first ICIs approved for second-line treatment of advanced/metastatic NSCLC after progression on platinum-based chemotherapy, based on the pivotal CheckMate 017 and CheckMate 057 phase III trials, which demonstrated significant survival benefit over docetaxel [4,5]; long-term follow-up of these trials has confirmed a durable survival advantage extending to five years [6,7]. It should be noted that the treatment landscape has since evolved further: ICI-based combinations, and in many settings ICI monotherapy or chemo-immunotherapy, are now frequently used in the first-line metastatic setting, whereas nivolumab monotherapy in NSCLC is used predominantly, though not exclusively, in the second-line setting today.

Despite this progress, the magnitude of clinical benefit from ICI therapy varies considerably among patients: while a subset achieves durable disease control, a substantial proportion experiences primary resistance and rapid clinical deterioration. Established predictive biomarkers, particularly tumor PD-L1 expression, are widely used [8,9] but are limited by tissue availability, sampling variability, and assay heterogeneity. Consequently, there is growing interest in inexpensive, readily reproducible, blood-based biomarkers reflecting the host systemic inflammatory and nutritional status that could complement existing predictive tools [10,11]. It is important to note at the outset that such inflammation-based scores are, by their nature, markers of the host–tumor systemic state and overall prognosis; they are not designed, and have not been shown, to predict the relative benefit of one treatment over another, a distinction we return to throughout this manuscript.

The modified Glasgow Prognostic Score (mGPS) is a simple scoring system derived from serum C-reactive protein (CRP) and albumin concentrations that reflects the magnitude of the systemic inflammatory response and nutritional reserve of the host [12]. Patients with CRP > 10 mg/L and albumin ≥ 35 g/L are classified as mGPS 1; those with both CRP > 10 mg/L and albumin < 35 g/L are classified as mGPS 2; and patients with CRP ≤ 10 mg/L are classified as mGPS 0 irrespective of albumin level [12,13]. Since its original description by Forrest and colleagues in patients with inoperable NSCLC [14], the mGPS has been validated as an independent prognostic factor across numerous solid tumors, including colorectal, gastric, renal cell, hepatocellular, and lung cancers, in both surgical and non-surgical settings [12,13,15,16].

An expanding body of literature has examined the prognostic role of mGPS specifically in patients with NSCLC treated with ICIs [17]. Recent meta-analyses have consistently reported that elevated mGPS is significantly associated with inferior OS and PFS in ICI-treated NSCLC populations [18,19], and a real-world study has directly compared mGPS with several other inflammation-based indices, including the neutrophil-to-lymphocyte ratio (NLR), in nivolumab-treated NSCLC [20]. Given this existing literature, the present study does not aim to establish mGPS as a novel biomarker; rather, its specific contribution is threefold. First, most prior cohorts pool heterogeneous ICI regimens and treatment lines, whereas this study focuses on a relatively homogeneous, single-agent, predominantly second-line nivolumab-treated population, reducing regimen-related heterogeneity as a potential confounder. Second, we formally test—rather than assume—whether mGPS provides prognostic information beyond NLR (and vice versa) using nested-model likelihood ratio testing, directly addressing whether these two inflammation-based indices are interchangeable or complementary in this setting. Third, we subject the primary findings to a battery of diagnostic and robustness analyses not uniformly reported in prior mGPS literature, including formal testing of the proportional hazards assumption, bootstrap-based testing of the incremental discrimination attributable to mGPS, a categorical (non-ordinal) sensitivity analysis, and a fully covariate-adjusted sensitivity analysis restricted to patients with known PD-L1 status.

The aim of this single-center retrospective cohort study was therefore to determine whether pretreatment mGPS is associated with PFS and OS, independent of other known clinical and laboratory prognostic factors measured in this cohort, in a relatively homogeneous, predominantly second-line, nivolumab-treated real-world population, and to rigorously characterize the robustness, model assumptions, and incremental value of this association through the sensitivity analyses outlined above.

## 2. Materials and Methods

### 2.1. Study Design and Patient Population

This was a retrospective, single-center cohort study conducted at the Department of Medical Oncology, Çukurova University Faculty of Medicine, Adana, Turkey. The final study cohort included 130 adult patients with histologically confirmed stage IV/metastatic NSCLC who received nivolumab (3 mg/kg every 2 weeks or a 240 mg fixed dose every 2 weeks, intravenously) and had available baseline CRP/albumin measurements and evaluable survival data. The majority of patients (97.7%, *n* = 127) had received prior platinum-based chemotherapy, and nivolumab was administered as second-line or later therapy in 96.9% (*n* = 126) of the cohort; four patients (3.1%) received nivolumab as first-line systemic therapy for metastatic disease. Because the cohort is predominantly (83.8%) second-line, the findings of this study should be interpreted as most directly applicable to that setting; extrapolation to first-line ICI or chemo-immunotherapy combination regimens, which now dominate front-line practice in many settings, requires separate validation (see Section 4).

Demographic characteristics (age, sex, smoking history), tumor histology, baseline ECOG performance status [21], PD-L1 tumor proportion score (TPS, where available), metastatic pattern (synchronous/de novo versus metachronous, presence of brain or liver metastasis), number of prior treatment lines before nivolumab, and baseline routine laboratory parameters (complete blood count, albumin, CRP, lactate dehydrogenase) were extracted retrospectively from electronic medical records using a standardized data collection form.

### 2.2. Definition of mGPS and NLR

The mGPS was calculated from serum CRP and albumin values obtained as part of the routine pretreatment laboratory panel at, or within the days immediately preceding, nivolumab initiation, in keeping with standard institutional clinical practice; the exact interval between laboratory sampling and treatment start was not separately captured as a discrete variable in the database, and mGPS was not re-measured on treatment. The standard mGPS definition was applied: patients with CRP ≤ 10 mg/L were classified as mGPS 0 regardless of albumin level; patients with CRP > 10 mg/L and albumin ≥ 35 g/L were classified as mGPS 1; and patients with both CRP > 10 mg/L and albumin < 35 g/L were classified as mGPS 2. The neutrophil-to-lymphocyte ratio (NLR), a widely studied blood-based prognostic index in ICI-treated cancer patients [10,11], was calculated as the absolute neutrophil count divided by the absolute lymphocyte count at baseline and dichotomized as high versus low using the cohort median (4.40) as the cut-off.

### 2.3. Study Endpoints

The study endpoints were PFS and OS. PFS was defined as the time from nivolumab initiation to radiological progression according to Response Evaluation Criteria in Solid Tumors (RECIST) version 1.1 [22], or death from any cause, whichever occurred first. OS was defined as the time from nivolumab initiation to death from any cause. Patients without a documented progression or death event were censored at the date of last follow-up.

### 2.4. Statistical Analysis

Continuous variables are presented as mean ± standard deviation (SD) or median (interquartile range [IQR]) according to distribution, and categorical variables as number (percentage). Categorical variables were compared across mGPS groups using the chi-square test; for variables with an expected cell count < 5 in one or more cells of the 2 × 3 table, a Monte Carlo estimate (200,000 permutations) of the Fisher–Freeman–Halton exact test was used instead. Continuous variables were compared using the Kruskal–Wallis test. PFS and OS were estimated using the Kaplan–Meier method, and differences among mGPS groups were assessed using the log-rank test. The effect of mGPS on PFS and OS was examined as an ordinal variable (0, 1, 2) in univariable and multivariable Cox proportional hazards regression models. The primary multivariable model included mGPS together with clinically relevant covariates, selected on the basis of established prognostic relevance in advanced NSCLC reported in prior clinicopathological and ICI-outcome literature rather than univariable screening in this dataset: age (≥65 vs. <65 years), sex, ECOG performance status (≥2 vs. <2), histology (non-squamous vs. squamous), brain metastasis, liver metastasis, and NLR (high vs. low). This pre-specification strategy, together with restriction to a limited, clinically justified covariate set, was intended to reduce the risk of overfitting given the number of observed events (69 for OS, 87 for PFS) relative to the number of candidate predictors; the corresponding events-per-variable ratio (approximately 8.6 for OS) is nonetheless modest and is discussed as a limitation. The proportional hazards assumption for each covariate in the primary models was formally assessed using scaled Schoenfeld residuals (Grambsch–Therneau test), with statistical tests and, where indicated, stratified sensitivity models used to evaluate covariates showing evidence of non-proportionality. To quantify whether mGPS and NLR provide overlapping or complementary prognostic information, nested Cox models (mGPS alone vs. mGPS+NLR, and NLR alone vs. NLR+mGPS) were compared using the likelihood ratio test. The incremental discriminative contribution of mGPS beyond the clinical covariates was assessed by comparing Harrell’s concordance index (C-index) between the clinical-variables-only model and the same model with mGPS added. Because a single-sample C-index comparison does not itself provide a measure of uncertainty, we additionally performed bootstrap inference for the paired difference in C-index (2000 iterations): in each iteration, both models were re-fitted on a bootstrap sample and evaluated on the original (non-resampled) cohort, yielding a bootstrap distribution of the paired C-index difference from which a 95% percentile confidence interval and a two-sided bootstrap *p*-value were derived. This procedure quantifies the statistical uncertainty of the observed within-cohort difference and is not a form of external or cross-validation; it does not substitute for, and should not be interpreted as, external validation of model discrimination in an independent cohort. As an exploratory sensitivity analysis, a Cox model incorporating mGPS together with all seven clinical covariates and PD-L1 status (≥1% vs. <1%) was additionally fitted in the subgroup of patients with available PD-L1 data; given the reduced number of events in this subgroup, this model is regarded as supportive rather than confirmatory. As a secondary, hypothesis-generating sensitivity analysis, backward elimination was additionally performed, in which mGPS was forced into the model while covariates with *p* > 0.10 were sequentially removed. A two-sided *p*-value < 0.05 was considered statistically significant. All analyses were performed using Python (version 3.11; Python Software Foundation, Wilmington, DE, USA) with the lifelines and SciPy packages.

## 3. Results

### 3.1. Patient Characteristics

A total of 130 patients were included in the final analysis. The mean age was 63.4 ± 8.3 years, and the majority of patients were male (*n* = 119, 91.5%). Most patients (69.2%, *n* = 90) had an ECOG performance status of 1 at baseline, 20.8% (*n* = 27) had ECOG 0, and 10.0% (*n* = 13) had ECOG ≥ 2. Adenocarcinoma (47.7%, *n* = 62) and squamous cell carcinoma (46.9%, *n* = 61) were the predominant histological subtypes. Metastatic disease was synchronous (de novo) in 66.2% (*n* = 86) and metachronous in 33.8% (*n* = 44) of patients; 20.0% (*n* = 26) had brain metastasis and 10.8% (*n* = 14) had liver metastasis at baseline. Nearly all patients (97.7%, *n* = 127) had received prior platinum-based chemotherapy, and nivolumab was administered as second-line therapy in 83.8% (*n* = 109) of cases. The distribution of pretreatment mGPS was 30.8% (*n* = 40) for mGPS 0, 42.3% (*n* = 55) for mGPS 1, and 26.9% (*n* = 35) for mGPS 2. The median potential follow-up, estimated using the reverse Kaplan–Meier method, was 14.9 months. At the data cutoff, death had occurred in 69 patients (53.1%) and a PFS event (progression or death) had occurred in 87 patients (66.9%). Detailed baseline characteristics are presented in Table 1.

### 3.2. Comparison of Baseline Characteristics Across mGPS Groups

Age distribution (Kruskal–Wallis *p* = 0.069) and ECOG performance status (*p* = 0.002, Monte Carlo exact test) showed borderline/significant differences across mGPS groups, with a higher proportion of ECOG ≥ 2 in the mGPS 1 and mGPS 2 groups compared with mGPS 0. As expected, NLR values differed significantly across mGPS groups (Kruskal–Wallis *p* < 0.001), with median NLR of 2.87, 4.50, and 8.09 in mGPS 0, 1, and 2, respectively, indicating that mGPS and NLR capture overlapping but not identical inflammatory information (Spearman r = 0.30). No statistically significant differences were observed across mGPS groups for sex, smoking history, histology, presence of brain or liver metastasis, or metastatic timing (all *p* > 0.05) (Table 2). Because ECOG performance status and NLR were both associated with mGPS group, and because ECOG remained non-significant while NLR fell short of significance in the fully adjusted model, residual confounding by these correlated, only partially overlapping measures of host fitness and systemic inflammation cannot be excluded and is discussed further below.

### 3.3. Survival Outcomes According to mGPS

**Figure 1:**
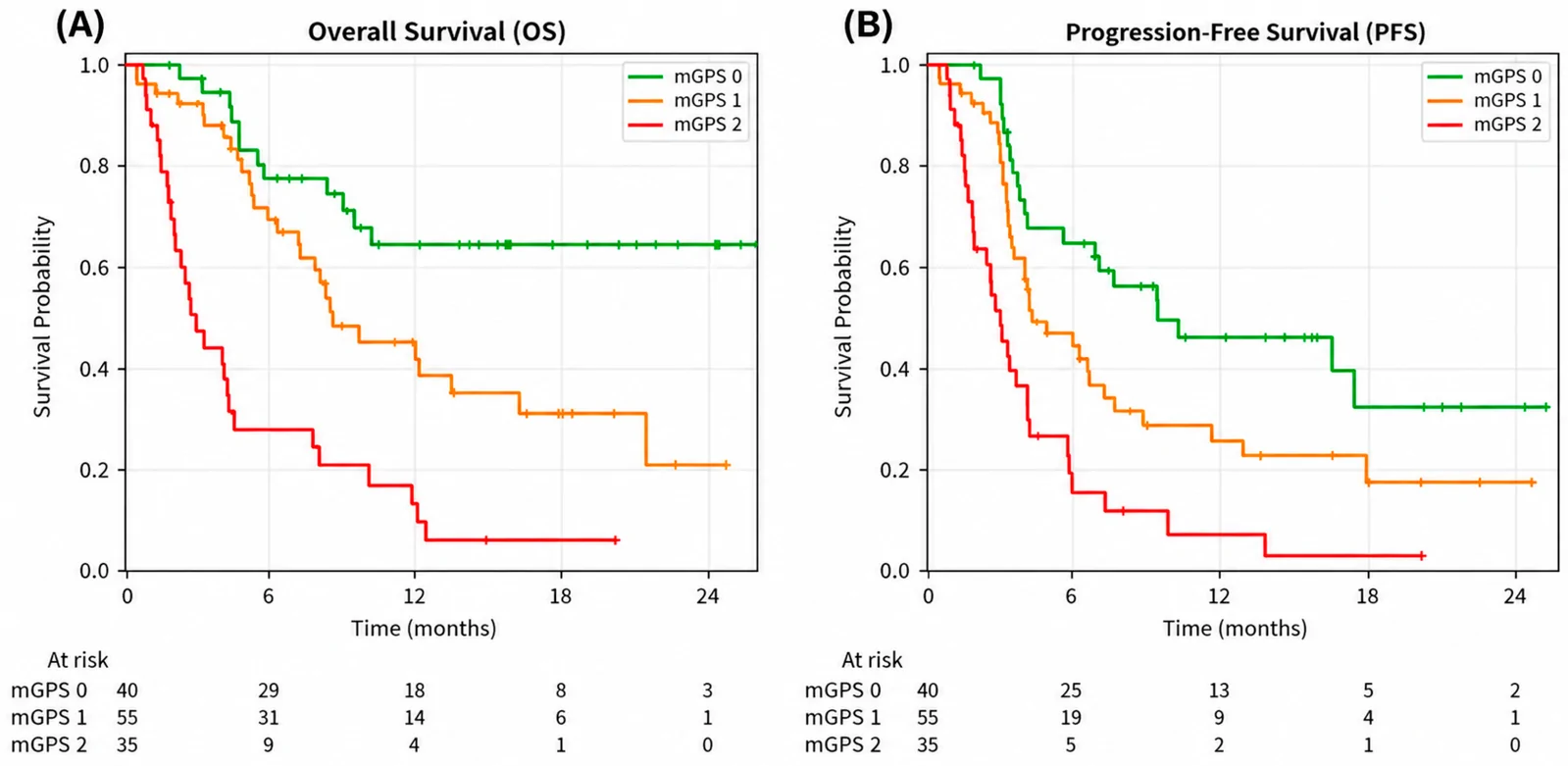
Kaplan–Meier curves for overall survival (**A**) and progression-free survival (**B**) according to pretreatment mGPS group (log-rank *p* < 0.001 for both endpoints). Tick marks (+) denote censored observations. Numbers at risk in each mGPS group at 0, 6, 12, 18, and 24 months are shown below each panel.

On Kaplan–Meier analysis, median OS was not reached, 9.7 months (approximate 95% CI 8.3–NA), and 3.0 months (approximate 95% CI 2.3–4.6) for the mGPS 0, mGPS 1, and mGPS 2 groups, respectively (log-rank *p* < 0.001). Median PFS was 10.2, 4.8, and 3.0 months, respectively (log-rank *p* < 0.001). The separation among mGPS groups was evident from early in the treatment course for both OS and PFS (Figure 1).

### 3.4. Univariable Cox Regression Analysis

On univariable Cox regression, each one-point increase in mGPS (0 → 1 → 2) was associated with a 2.69-fold increased risk of death (95% CI 1.91–3.78; *p* < 0.001) and a 1.95-fold increased risk of progression or death (95% CI 1.46–2.60; *p* < 0.001). High NLR was also significantly associated with both OS (HR 2.20; 95% CI 1.36–3.57; *p* = 0.001) and PFS (HR 1.62; 95% CI 1.06–2.48; *p* = 0.025). Age ≥ 65 years showed a borderline protective association with OS (HR 0.61; 95% CI 0.37–0.99; *p* = 0.048); non-squamous histology showed a borderline association with OS (HR 1.55; 95% CI 0.96–2.51; *p* = 0.071). Sex, ECOG performance status, brain metastasis, liver metastasis, and metastatic timing were not significantly associated with either endpoint on univariable analysis (Table 3).

### 3.5. Multivariable Cox Regression Analysis

To assess the prognostic value of mGPS independent of other measured covariates, multivariable Cox models were constructed adjusting for age (≥65 vs. <65 years), sex, ECOG performance status (≥2 vs. <2), histology (non-squamous vs. squamous), brain metastasis, liver metastasis, and NLR (high vs. low). Given the number of observed events (69 for OS, 87 for PFS) relative to the eight covariates included, the model was kept intentionally parsimonious, restricted to established clinical variables and mGPS; the corresponding events-per-variable ratio (approximately 8.6 for OS) should be considered when interpreting the precision of individual covariate estimates, and is addressed further in the Limitations. In this primary multivariable model, mGPS remained significantly associated with both OS (adjusted HR 2.38; 95% CI 1.64–3.46; *p* < 0.001; Harrell’s concordance index = 0.737) and PFS (adjusted HR 1.81; 95% CI 1.32–2.48; *p* < 0.001; Harrell’s concordance index = 0.666) (Table 4), independent of the specific covariates included in this model; independence from unmeasured or unadjusted factors cannot be inferred and is not claimed. None of the other covariates included in the model reached statistical significance when evaluated alongside mGPS (all *p* > 0.05), although age showed a borderline trend for OS (*p* = 0.095).

### 3.6. Proportional Hazards Assumption and Nested-Model Comparison of mGPS and NLR

The proportional hazards assumption for the primary multivariable models was assessed using scaled Schoenfeld residuals for each covariate. For mGPS, the variable of primary interest, there was no evidence of violation of the proportional hazards assumption in either the OS model (*p* = 0.51) or the PFS model. In the OS model only, two covariates showed evidence of non-proportional hazards on formal testing: brain metastasis (*p* = 0.028) and high NLR (*p* = 0.047); no violations were detected in the PFS model. As a sensitivity analysis, the OS model was refitted stratified by brain metastasis and NLR category (removing the proportionality constraint for these two variables); the mGPS estimate was materially unchanged (stratified adjusted HR 2.40, 95% CI 1.62–3.56, *p* < 0.001, versus 2.38, 95% CI 1.64–3.46, *p* < 0.001 in the original model), indicating that the primary conclusion regarding mGPS is not driven by, or sensitive to, the non-proportionality detected in these secondary covariates.

To directly test whether mGPS and NLR provide overlapping or complementary prognostic information, nested Cox models were compared using the likelihood ratio (LR) test. Adding NLR to a model already containing mGPS did not significantly improve model fit for either endpoint (OS: LR χ^2^ = 1.49, *p* = 0.222; PFS: LR χ^2^ = 0.28, *p* = 0.597). In contrast, adding mGPS to a model already containing NLR significantly improved model fit for both endpoints (OS: LR χ^2^ = 25.19, *p* < 0.001; PFS: LR χ^2^ = 16.02, *p* < 0.001). These findings suggest that, in this cohort, mGPS provided incremental prognostic information beyond NLR, whereas NLR did not provide statistically significant incremental information once mGPS was included; this is consistent with the multivariable finding that NLR was not independently significant once mGPS was included (Table 4). We note that this asymmetry describes the statistical behavior of these two markers in nested Cox models in our data and should not be over-interpreted as evidence that mGPS causally subsumes the biological information reflected by NLR.

### 3.7. Incremental Discrimination and Bootstrap Testing

To quantify the incremental prognostic contribution of mGPS beyond established clinical variables, Harrell’s concordance index was compared between a clinical-variables-only model (age, sex, ECOG performance status, histology, brain metastasis, liver metastasis, and NLR) and the same model with mGPS added. Addition of mGPS was associated with a higher C-index for both endpoints, from 0.676 to 0.737 for OS (observed difference +0.061) and from 0.620 to 0.666 for PFS (observed difference +0.047) (Table 5). To quantify the statistical uncertainty of this observed difference, we performed bootstrap inference for the paired difference in C-index (2000 iterations), in which both models were re-fitted on each bootstrap sample and evaluated on the original cohort; this bootstrap procedure is used here strictly as a method of statistical inference on the magnitude and uncertainty of the within-cohort difference, and not as a form of external or cross-validation. This analysis indicated that the observed difference was unlikely to be due to chance alone, for both OS (bootstrap mean difference +0.063, 95% CI 0.038–0.092, *p* < 0.001) and PFS (bootstrap mean difference +0.055, 95% CI 0.030–0.080, *p* < 0.001), with 95% confidence intervals excluding zero for both endpoints. This provides formal statistical support, beyond the previously descriptive single-sample comparison, that mGPS improves discrimination over the clinical-variables-only model within this cohort; because this is an internal (in-sample) estimate from a single-center cohort, external validation in an independent dataset remains necessary before the magnitude of this improvement can be generalized.

### 3.8. Categorical mGPS, PD-L1-Adjusted, and Backward-Elimination Sensitivity Analyses

Because mGPS was modeled as an ordinal continuous variable (0, 1, 2) in the primary analysis, a categorical sensitivity analysis was performed using mGPS 0 as the reference category to examine whether the assumption of a linear per-unit effect was reasonable. On univariable analysis, mGPS 1 and mGPS 2 were associated with progressively increasing risk relative to mGPS 0 for both OS (HR 2.27, 95% CI 1.15–4.48, *p* = 0.018 for mGPS 1; HR 6.85, 95% CI 3.45–13.59, *p* < 0.001 for mGPS 2) and PFS (HR 1.75, 95% CI 1.02–3.01, *p* = 0.042 for mGPS 1; HR 3.74, 95% CI 2.12–6.59, *p* < 0.001 for mGPS 2), a pattern that remained directionally consistent after adjustment for the same clinical covariates as in Table 4 (adjusted HR for mGPS 1 vs. 0: 1.87, 95% CI 0.91–3.84, *p* = 0.088 for OS, and 1.63, 95% CI 0.91–2.94, *p* = 0.103 for PFS; adjusted HR for mGPS 2 vs. 0: 5.31, 95% CI 2.54–11.09, *p* < 0.001 for OS, and 3.23, 95% CI 1.72–6.06, *p* < 0.001 for PFS), supporting the ordinal coding used in the primary analysis. Although the adjusted estimates for mGPS 1 did not reach statistical significance for either endpoint, point estimates increased monotonically across mGPS 0 → 1 → 2, consistent with the ordinal (linear per-unit) modeling approach used in the primary analysis.

As tumor PD-L1 expression was not available for the full cohort, two complementary PD-L1 sensitivity analyses were performed in the subgroup of 92 patients (70.8%) with available PD-L1 data. First, an exploratory two-variable Cox model including only mGPS and PD-L1 status (≥1% vs. <1%) showed that mGPS remained significantly associated with both OS (HR 2.85, 95% CI 1.87–4.33, *p* < 0.001) and PFS (HR 2.09, 95% CI 1.47–2.98, *p* < 0.001), while PD-L1 status was not independently associated with either endpoint (OS *p* = 0.744; PFS *p* = 0.836); a similar pattern was observed using a ≥50% PD-L1 cutoff. Second, and addressing the possibility that this simplified two-variable model masked confounding by the other clinical covariates, an additional exploratory sensitivity model—the full primary multivariable model (mGPS plus all seven clinical covariates in Table 4) refitted with PD-L1 status added—was fitted in the same 92-patient subgroup; we emphasize that, with only 48 OS and 62 PFS events available in this subgroup relative to nine covariates, this exploratory model is more susceptible to overfitting than the two-variable model above and than the primary model in the full cohort, and its point estimates should be interpreted with corresponding caution rather than as a confirmatory analysis. In this exploratory, fully covariate-adjusted model, mGPS remained strongly associated with both OS (adjusted HR 2.70, 95% CI 1.61–4.51, *p* < 0.001) and PFS (adjusted HR 2.34, 95% CI 1.51–3.63, *p* < 0.001), while PD-L1 status remained non-significant (OS HR 1.25, *p* = 0.472; PFS HR 1.10, *p* = 0.728); the mGPS estimate in this subgroup was materially unchanged whether or not PD-L1 was included in the model (OS: 2.67 without vs. 2.70 with PD-L1; PFS: 2.33 without vs. 2.34 with PD-L1). Taken together, these two exploratory sensitivity analyses suggest that the association between mGPS and survival is not obviously attributable to confounding by PD-L1 status in the subgroup with available data, although the possibility of differential associations in the 29.2% of patients with missing PD-L1 cannot be excluded, and neither analysis should be regarded as a substitute for the primary model in Table 4.

As a secondary, hypothesis-generating check, the backward-elimination analysis described in Methods retained mGPS as the only variable in the final model for both OS (HR 2.69, 95% CI 1.91–3.78, *p* < 0.001) and PFS (HR 1.95, 95% CI 1.46–2.60, *p* < 0.001); given the modest sample size, this data-driven result is considered supportive rather than primary evidence, with the primary multivariable model (Table 4) remaining the main evidence for the mGPS-survival association. A summary of all sensitivity and robustness analyses performed is presented in Table 6.

## 4. Discussion

In this single-center retrospective cohort study, pretreatment mGPS was significantly associated with both OS and PFS in patients with advanced/metastatic NSCLC treated with nivolumab, independent of the age, sex, ECOG performance status, histology, brain/liver metastasis, and NLR measured in this cohort. The progressive decline in median OS from not-reached to 3.0 months, and in median PFS from 10.2 to 3.0 months, as mGPS increased from 0 to 2, demonstrates clear prognostic stratification across mGPS categories. We emphasize at the outset that these findings support mGPS as a prognostic marker—informative of the likely disease trajectory of a patient about to start nivolumab—and not as a predictive biomarker of the relative benefit of nivolumab over alternative treatments; no comparator arm or treatment-by-biomarker interaction was tested in this single-arm cohort, and such a claim would not be supported by this design.

The prognostic value of mGPS in ICI-treated NSCLC populations has previously been reported—including a recent meta-analysis of 11 studies comprising 1022 patients showing that elevated mGPS was significantly associated with inferior OS and PFS [18], and a real-world study directly comparing seven inflammation-based indices, including mGPS, in nivolumab-treated advanced NSCLC [20]. Rather than proposing mGPS as a new biomarker, the present study extends this evidence in three specific ways. First, it focuses on a relatively homogeneous, predominantly second-line (83.8%), single-agent nivolumab-treated real-world cohort, reducing regimen- and line-of-therapy-related heterogeneity as a source of confounding relative to pooled multi-regimen cohorts. Second, we directly and formally tested whether NLR provides prognostic information beyond mGPS, and vice versa, using nested-model likelihood ratio testing rather than simply including both markers in a single model and noting which remained significant: these findings suggest that, in this cohort, mGPS provided incremental prognostic information beyond NLR, whereas NLR did not provide statistically significant incremental information once mGPS was included (addition of NLR to mGPS did not improve model fit, *p* = 0.22–0.60; addition of mGPS to NLR did, *p* < 0.001). mGPS provided greater incremental prognostic information than NLR in this cohort, though this finding requires replication before being generalized. Third, the primary association was subjected to a battery of diagnostic and robustness analyses—proportional hazards testing, bootstrap-based testing of incremental discrimination, categorical (non-ordinal) modeling, and a fully covariate-adjusted PD-L1 sensitivity analysis—that are not uniformly reported in the existing mGPS-ICI literature and that, taken together, argue against the primary finding being an artifact of model specification, non-proportionality, or unadjusted PD-L1 confounding within the limits of the data available.

Our findings are consistent with the emerging literature supporting the prognostic value of mGPS in ICI-treated NSCLC populations. Tanaka and colleagues reported that elevated mGPS was significantly associated with shorter PFS and OS in elderly patients with advanced NSCLC treated with anti-PD-1 antibodies [17]. Recent meta-analyses pooling data from ICI-treated NSCLC cohorts have shown that elevated mGPS is associated with significantly increased risk for both OS and PFS [18,19], with pooled hazard ratios (approximately 1.6–1.7) that are directionally consistent with, though numerically lower than, the univariable per-unit estimates observed in our cohort (OS HR 2.69 and PFS HR 1.95 per one-point mGPS increase); this difference may partly reflect the use of a per-unit ordinal HR in our analysis versus pooled dichotomized (high vs. low mGPS) estimates in most meta-analyses, as well as differences in patient populations and ICI regimens across pooled studies. The prognostic value of preoperative mGPS has also been demonstrated in surgically resected NSCLC [15] and in patients treated with stereotactic body radiotherapy [16], suggesting that mGPS may reflect a treatment-modality-independent marker of host–tumor interaction and tumor biology rather than an ICI-specific phenomenon.

A plausible, though not directly tested, biological rationale for the prognostic value of mGPS relates to the interplay between chronic systemic inflammation, tumor progression, and immune evasion [12,13]. Elevated CRP reflects an interleukin-6-driven acute-phase response that has, in other experimental and translational studies not conducted as part of the present work, been associated with expansion of myeloid-derived suppressor cells and M2-polarized tumor-associated macrophages within the tumor microenvironment, potentially suppressing cytotoxic T-lymphocyte function and blunting the efficacy of PD-1 blockade; we did not measure any immune cell subsets, cytokines, or tumor microenvironment parameters in this cohort, and these mechanisms should be regarded as a hypothesis-generating interpretive framework rather than a finding of this study. Similarly, hypoalbuminemia is generally understood to reflect both malnutrition and systemic inflammation and has been associated with sarcopenia and reduced functional reserve in other cohorts [23,24]; whether this pathway contributed to the associations observed here cannot be determined from the available data. The moderate correlation observed between mGPS and NLR in our cohort (r = 0.30) indicates that these two markers capture overlapping but not identical inflammatory information [10,11,25], consistent with the likelihood-ratio-test finding above and with observations from other nivolumab-treated NSCLC cohorts comparing multiple immune-inflammatory indices [20,26,27,28] and with systematic reviews and meta-analyses supporting the prognostic utility of NLR-based indices in ICI-treated cancer populations more broadly [29].

Because mGPS is partly constituted by, and correlated with, other markers of host fitness and systemic inflammation—including ECOG performance status and NLR, both of which differed significantly across mGPS groups in this cohort (Table 2)—residual confounding by these and other unmeasured indicators of systemic inflammation or nutritional status (e.g., body composition, cytokine profiles, sarcopenia measures) cannot be excluded despite statistical adjustment for ECOG and NLR in the primary model. The description of mGPS’s association with survival as “independent” throughout this manuscript should accordingly be understood specifically as independence from the covariates measured and included in our models (age, sex, ECOG, histology, brain/liver metastasis, and NLR), and not as independence from all conceivable prognostic confounders, a stronger claim that this observational, single-center dataset cannot support.

The borderline protective association observed between age ≥ 65 years and OS on univariable analysis (HR 0.61, *p* = 0.048) is somewhat counterintuitive but may reflect selection: in routine clinical practice, older patients selected for nivolumab therapy may represent a fitter subgroup with fewer comorbidities and better baseline performance status (‘healthy elderly’ selection bias); this association lost statistical significance in the multivariable model (*p* = 0.095) and should therefore be interpreted with caution.

A key strength of this study is its evaluation of mGPS’s prognostic value in a primary multivariable model incorporating multiple clinically relevant covariates commonly used in routine practice, together with formal diagnostic testing (proportional hazards assumption) and bootstrap-based significance testing of the incremental discrimination attributable to mGPS—an analysis that, to our knowledge, has not been uniformly applied in the prior mGPS-ICI literature and that provides formal statistical inference regarding the observed within-cohort difference in discrimination. The backward-elimination sensitivity analysis further supports the robustness of the mGPS-survival association, independent of model specification, although we regard this data-driven procedure as secondary to, rather than a replacement for, the pre-specified primary model.

This study has several limitations that should inform the interpretation of these findings. First, its retrospective, single-center, observational design cannot exclude the influence of unmeasured confounding factors, and external validation in independent, ideally multicenter, cohorts is required before these findings can be generalized. Second, the relatively modest sample size (*n* = 130) and number of events (69 for OS, 87 for PFS) relative to the eight covariates in the primary model (events-per-variable ratio of approximately 8.6 for OS) create a non-trivial risk of overfitting; we sought to mitigate this through a priori clinical covariate selection rather than univariable screening, a parsimonious covariate set, and multiple sensitivity analyses (categorical modeling, backward elimination, stratified and PD-L1-adjusted models) that were broadly consistent with the primary estimates, but this risk cannot be eliminated in a dataset of this size. Third, as detailed above, mGPS is correlated with ECOG performance status and NLR, and residual confounding by these or other unmeasured indicators of systemic inflammation and nutritional status cannot be excluded despite adjustment; the term “independent” in this manuscript refers specifically to independence from the measured covariates included in our models. Fourth, PD-L1 expression data were available in only 70.8% of patients; although two complementary sensitivity analyses restricted to this subgroup did not suggest confounding by PD-L1 status, these analyses were themselves limited by reduced sample size, and residual uncertainty regarding the missing 29.2% remains. Fifth, this cohort was predominantly (83.8%) second-line, single-agent nivolumab monotherapy; because first-line treatment of advanced NSCLC now frequently involves ICI-based combinations or chemo-immunotherapy in many practice settings, these findings should not be assumed to generalize to first-line ICI or combination regimens without separate validation. Sixth, as a single-center study, generalizability to other populations and practice settings may be further limited. Finally, the observed improvement in model discrimination with mGPS, while now formally tested and statistically significant via bootstrap resampling, was modest in absolute magnitude and was derived from internal (in-sample) model evaluation; it does not by itself establish clinical utility (e.g., in terms of changed clinical decisions or patient-relevant outcomes), which would require prospective evaluation, ideally with external validation of model discrimination. Prospective, adequately powered, multicenter studies—including in first-line ICI settings—are needed to confirm and extend these findings.

## 5. Conclusions

In this cohort, pretreatment mGPS was significantly associated with both PFS and OS in patients with advanced/metastatic NSCLC treated predominantly with second-line nivolumab monotherapy, independent of the clinical covariates measured and included in our models. Its addition to established clinical variables was associated with a statistically significant, though modest, improvement in prognostic discrimination, confirmed using bootstrap resampling rather than descriptive comparison alone. As a prognostic (rather than predictive) marker, mGPS reflects the likely disease trajectory of a patient starting nivolumab rather than the relative benefit of nivolumab versus alternative treatments; it should not be used to withhold ICI therapy or to infer differential treatment benefit, and its independence should be understood as independence from the specific covariates adjusted for in this study rather than from all possible confounders. Given the single-center design, the predominance of second-line patients, and the modest sample size relative to the covariates modeled, these findings should be regarded as hypothesis-supporting rather than practice-changing. Prospective, multicenter validation—including in first-line immunotherapy settings and with more complete PD-L1 ascertainment—and integration of mGPS with other biomarkers (e.g., PD-L1 expression, tumor mutational burden) in combined prognostic models are recommended before mGPS can be recommended for routine clinical risk stratification.

## Figures and Tables

**Table 1:** Baseline demographic, clinical, and laboratory characteristics of the study cohort (*n* = 130).

Variable	*n* (%)/Mean ± SD/Median (IQR)
Age, mean ± SD (years)	63.4 ± 8.3
Age ≥ 65 years, *n* (%)	61 (46.9%)
Sex, Male, *n* (%)	119 (91.5%)
Sex, Female, *n* (%)	11 (8.5%)
Smoking history, Never, *n* (%)	15 (11.5%)
Smoking history, Ever (current/former), *n* (%)	115 (88.5%)
ECOG PS 0, *n* (%)	27 (20.8%)
ECOG PS 1, *n* (%)	90 (69.2%)
ECOG PS ≥ 2, *n* (%)	13 (10.0%)
Histology, Adenocarcinoma, *n* (%)	62 (47.7%)
Histology, Squamous cell carcinoma, *n* (%)	61 (46.9%)
Histology, Other, *n* (%)	7 (5.4%)
PD-L1 TPS < 1%, *n*/*N* (%) *	40/92 (43.5%)
PD-L1 TPS 1–49%, *n*/*N* (%) *	30/92 (32.6%)
PD-L1 TPS ≥ 50%, *n*/*N* (%) *	22/92 (23.9%)
Metastatic timing, De novo (synchronous), *n* (%)	86 (66.2%)
Metastatic timing, Metachronous, *n* (%)	44 (33.8%)
Brain metastasis, Present, *n* (%)	26 (20.0%)
Liver metastasis, Present, *n* (%)	14 (10.8%)
Prior platinum-based chemotherapy, *n* (%)	127 (97.7%)
Nivolumab treatment line, 1st line, *n* (%)	4 (3.1%)
Nivolumab treatment line, 2nd line, *n* (%)	109 (83.8%)
Nivolumab treatment line, ≥3rd line, *n* (%)	17 (13.1%)
NLR, median (IQR)	4.40 (2.30–6.75)
mGPS 0, *n* (%)	40 (30.8%)
mGPS 1, *n* (%)	55 (42.3%)
mGPS 2, *n* (%)	35 (26.9%)
OS event (death), *n* (%)	69 (53.1%)
PFS event (progression/death), *n* (%)	87 (66.9%)
Median potential follow-up, months (reverse KM)	14.9

SD: standard deviation; IQR: interquartile range; ECOG PS: Eastern Cooperative Oncology Group performance status; PD-L1 TPS: programmed death-ligand 1 tumor proportion score; NLR: neutrophil-to-lymphocyte ratio; mGPS: modified Glasgow Prognostic Score; OS: overall survival; PFS: progression-free survival; KM: Kaplan–Meier. * PD-L1 TPS was available in 92 patients (70.8%); data were missing in 38 patients (29.2%).

**Table 2:** Distribution of selected clinical variables according to mGPS group.

Variable	mGPS 0 (*n* = 40)	mGPS 1 (*n* = 55)	mGPS 2 (*n* = 35)	*p*
Age ≥ 65 years, *n* (%)	19 (47.5%)	31 (56.4%)	11 (31.4%)	0.069
Female sex, *n* (%)	4 (10.0%)	5 (9.1%)	2 (5.7%)	0.852 ^†^
ECOG PS ≥ 2, *n* (%)	0 (0.0%)	11 (20.0%)	2 (5.7%)	0.002 ^†^
Non-squamous histology, *n* (%)	18 (45.0%)	29 (52.7%)	21 (60.0%)	0.429
Smoking history (ever), *n* (%)	36 (90.0%)	49 (89.1%)	30 (85.7%)	0.883 ^†^
Brain metastasis, *n* (%)	6 (15.0%)	11 (20.0%)	9 (25.7%)	0.512
Liver metastasis, *n* (%)	5 (12.5%)	3 (5.5%)	6 (17.1%)	0.215 ^†^
Metachronous metastasis, *n* (%)	15 (37.5%)	20 (36.4%)	9 (25.7%)	0.490
High NLR (>median), *n* (%)	9 (22.5%)	29 (52.7%)	27 (77.1%)	<0.001

Categorical variables were compared using the chi-square test. ^†^ For variables with an expected cell count < 5 in one or more cells (female sex, ECOG PS ≥ 2, smoking history, liver metastasis), a Monte Carlo estimate (200,000 permutations) of the Fisher–Freeman–Halton exact test was used instead, given the limitations of the asymptotic chi-square approximation in sparse 2 × 3 tables; results were consistent with the asymptotic chi-square test for all variables. mGPS: modified Glasgow Prognostic Score; ECOG PS: performance status; NLR: neutrophil-to-lymphocyte ratio.

**Table 3:** Univariable Cox regression analysis for overall survival (OS) and progression-free survival (PFS).

Variable	OS HR (95% CI)	*p*	PFS HR (95% CI)	*p*
mGPS (0 → 1 → 2, per unit)	2.69 (1.91–3.78)	<0.001	1.95 (1.46–2.60)	<0.001
Age ≥ 65 years (vs. <65)	0.61 (0.37–0.99)	0.048	0.67 (0.43–1.03)	0.067
Female sex (vs. male)	1.06 (0.48–2.32)	0.884	1.20 (0.60–2.39)	0.608
ECOG PS ≥ 2 (vs. <2)	1.60 (0.73–3.52)	0.239	1.36 (0.66–2.83)	0.405
Non-squamous histology	1.55 (0.96–2.51)	0.071	1.26 (0.82–1.92)	0.291
Brain metastasis (present)	1.28 (0.72–2.26)	0.405	1.33 (0.80–2.21)	0.271
Liver metastasis (present)	1.48 (0.73–2.98)	0.277	1.53 (0.83–2.82)	0.171
Metachronous metastasis	0.96 (0.57–1.60)	0.870	0.77 (0.48–1.23)	0.273
High NLR (>median)	2.20 (1.36–3.57)	0.001	1.62 (1.06–2.48)	0.025

HR: hazard ratio; CI: confidence interval; mGPS was modeled as an ordinal variable (0,1,2); ECOG PS: performance status; NLR: neutrophil-to-lymphocyte ratio.

**Table 4:** Primary multivariable Cox regression analysis for overall survival (OS) and progression-free survival (PFS).

Variable	OS Adjusted HR (95% CI)	*p*	PFS Adjusted HR (95% CI)	*p*
mGPS (0 → 1 → 2, per unit)	2.38 (1.64–3.46)	<0.001	1.81 (1.32–2.48)	<0.001
Age ≥ 65 years	0.62 (0.36–1.09)	0.095	0.64 (0.40–1.03)	0.067
Female sex	1.75 (0.77–3.98)	0.183	1.70 (0.82–3.50)	0.151
ECOG PS ≥ 2	2.00 (0.82–4.84)	0.126	1.50 (0.67–3.35)	0.320
Non-squamous histology	1.31 (0.77–2.24)	0.316	1.04 (0.65–1.66)	0.870
Brain metastasis (present)	1.40 (0.75–2.62)	0.285	1.30 (0.75–2.24)	0.355
Liver metastasis (present)	1.28 (0.62–2.67)	0.504	1.50 (0.79–2.84)	0.211
High NLR (>median)	1.48 (0.87–2.51)	0.145	1.18 (0.74–1.89)	0.486

The model was simultaneously adjusted for mGPS, age, sex, ECOG PS, histology, brain metastasis, liver metastasis, and NLR. HR: hazard ratio; CI: confidence interval. Harrell’s concordance index: 0.737 for the OS model and 0.666 for the PFS model.

**Table 5:** Model discrimination (Harrell’s C-index) with and without mGPS, including bootstrap-based significance testing.

Model/Comparison	OS	PFS
Clinical variables only (age, sex, ECOG PS, histology, brain met, liver met, NLR)	0.676	0.620
Clinical variables + mGPS	0.737	0.666
Observed C-index difference	+0.061	+0.047
Bootstrap mean difference (2000 resamples)	+0.063	+0.055
Bootstrap 95% CI for difference	0.038 to 0.092	0.030 to 0.080
Bootstrap two-sided *p*-value	<0.001	<0.001

ECOG PS: performance status; NLR: neutrophil-to-lymphocyte ratio; mGPS: modified Glasgow Prognostic Score. Bootstrap procedure: Both models were re-fitted on 2000 bootstrap resamples (sampling with replacement, *n* = 130 per resample) and evaluated on the original, non-resampled cohort to avoid tie-related bias from duplicated observations; the 95% CI is the 2.5th–97.5th percentile of the resulting bootstrap distribution of the C-index difference.

**Table 6:** Summary of sensitivity, robustness, and model-diagnostic analyses.

Analysis	Key Result
Proportional hazards assumption (mGPS)	Satisfied in both OS (*p* = 0.51) and PFS models
Proportional hazards assumption (other covariates)	OS model: violated for brain metastasis (*p* = 0.028) and high NLR (*p* = 0.047); PFS model: no violations
OS model stratified by brain metastasis and NLR	mGPS aHR 2.40 (95% CI 1.62–3.56), *p* < 0.001 (vs. 2.38 in primary model)
Categorical mGPS (0 = ref), adjusted	mGPS 2 vs. 0: OS aHR 5.31, PFS aHR 3.23 (both *p* < 0.001); mGPS 1 vs. 0: OS aHR 1.87 (*p* = 0.088), PFS aHR 1.63 (*p* = 0.103)
LR test: NLR added to mGPS	OS *p* = 0.222; PFS *p* = 0.597 (not significant)
LR test: mGPS added to NLR	OS *p* < 0.001; PFS *p* < 0.001 (significant)
PD-L1 two-variable model (*n* = 92)	mGPS aHR: OS 2.85, PFS 2.09 (both *p* < 0.001); PD-L1 not significant
PD-L1 full covariate-adjusted model (*n* = 92, exploratory)	mGPS aHR: OS 2.70, PFS 2.34 (both *p* < 0.001); PD-L1 not significant
Bootstrap C-index difference (mGPS added)	OS +0.063 (95% CI 0.038–0.092), *p* < 0.001; PFS +0.055 (95% CI 0.030–0.080), *p* < 0.001
Backward elimination	mGPS retained as sole variable for both OS and PFS (*p* < 0.001)

aHR: adjusted hazard ratio; LR: likelihood ratio; OS: overall survival; PFS: progression-free survival; mGPS: modified Glasgow Prognostic Score; NLR: neutrophil-to-lymphocyte ratio.

## Data Availability

The data presented in this study are available on request from the corresponding author due to institutional data-sharing restrictions on patient-level clinical data.
